# Ultrasound detection of normal parathyroid glands: detection rate, topographic anatomy, and the role of underlying thyroid disease

**DOI:** 10.3389/fendo.2025.1595940

**Published:** 2025-07-10

**Authors:** Isabella Chiardi, Petra Makovac, Andrea Leoncini, Flavio Forte, Mario Rotondi, Pierpaolo Trimboli

**Affiliations:** ^1^ Department of Internal Medicine and Therapeutics, University of Pavia, Pavia, Italy; ^2^ Thyroid Unit, Clinic for Endocrinology and Diabetology, Ente Ospedaliero Cantonale (EOC), Bellinzona, Switzerland; ^3^ Service of Surgery, Ospedale Regionale di Mendrisio, Ente Ospedaliero Cantonale (EOC), Mendrisio, Switzerland; ^4^ Clinic for Radiology, Imaging Institute of Southern Switzerland, Ente Ospedaliero Cantonale (EOC), Bellinzona, Switzerland; ^5^ Urologic Division, Vannini Hospital, Rome, Italy; ^6^ Istituti Clinici Scientifici Maugeri IRCCS, Unit of Endocrinology and Metabolism, Laboratory for Endocrine Disruptors, Pavia, Italy; ^7^ Faculty of Biomedical Sciences, Università Della Svizzera Italiana (USI), Lugano, Switzerland

**Keywords:** US, normal parathyroids, parathyroid detection, parathyroid imaging, neck ultrasound

## Abstract

**Introduction:**

Visualizing normal parathyroid glands (PTGs) using ultrasound (US) has historically been challenging. This study aims to assess the detection rate of normal PTGs in thyroid patients and evaluate their echostructure, anatomical location and their relation with the underlying thyroid pathology.

**Methods:**

A retrospective observational study was conducted over four weeks (September–October 2024) at the Thyroid Unit of Ente Ospedaliero Cantonale (EOC). Consecutive thyroid patients undergoing US for any thyroid indication were included, while those with a history of parathyroid disease, chronic kidney disease, or recent thyroid surgery were excluded. The primary outcome was the detection rate of normal PTGs. Secondary outcomes included PTG echostructure, anatomical location, and correlations with patient characteristics (age, gender, BMI, thyroid volume, and underlying thyroid pathology).

**Results:**

Normal PTGs were identified in 45.1% of patients (n=51). Most PTGs were located near the lower pole of the thyroid lobes and appeared mildly hyperechoic. Thyroid volume was inversely associated with PTG detection (p=0.001), while underlying thyroid pathology (e.g., thyroiditis, nodular disease) had no significant impact on detection rates.

**Conclusion:**

Normal PTGs can be visualized using US, particularly near the lower thyroid poles. Detection rates decrease in patients with larger thyroid volumes or athyreotic status. These findings confirm and expand on recent studies, challenging the historical belief that normal PTGs are undetectable, with potential implications for endocrine imaging and surgical planning.

## Introduction

Ultrasound (US) has traditionally been used to assess pathological parathyroid glands (PTGs) in hyperparathyroidism, with reported sensitivities ranging from 72% to 89% (1). However, for many years, it has been widely believed that normal PTGs were ultrasonographically not visible. This assumption derives from the fact that in the past US technology had lower sensitivity. Moreover, normal PTGs were rarely studied due to the absence of established criteria to help clinicians identify them. Recently, studies have explored the possibility of identifying normal PTGs on US and reported interesting results (2, 3). In general, these studies have described normal PTGs as small, with oval shape, and with homogeneous, mildly hyperechoic appearance. In some cases, authors (4) further validated the identification of normal PTGs by using fine-needle aspiration (FNA) and measuring parathyroid hormone (PTH) levels in FNA washout fluids, confirming the US findings. Recognizing that normal PTGs can indeed be visualized on US, and defining their US presentation, can be significant for clinical reasons. First, it allows to discriminate PTGs from metastatic lymph nodes, thereby avoiding unnecessary FNAs (3). Second, it helps in preoperative planning for thyroidectomy by improving the identification of PTGs, thereby preventing as much as possible a postoperative hypoparathyroidism. Despite these advancements, there is still limited data on the detection rates and localization of normal PTGs in patients with various thyroid disorders.

Therefore, the primary aim of this study was to analyze the detection rate of normal PTGs in a consecutive series of thyroid patients. In addition, echostructure and localization of PTGs were systematically investigated.

## Materials and methods

### Institutional setting

The Thyroid Unit of the Ente Ospedaliero Cantonale (EOC) serves as public referral center for thyroid diseases. Patients with any form of thyroid disorder are addressed to this unit. The setting is equipped with modern US machines (Siemens ACUSON NX3™ with 16 MHz linear transducer), enabling comprehensive monitoring and follow-up of patients.

### Case selection

According to the study aim, consecutive thyroid patients visited in a four-week period in autumn 2024 were initially screened and then evaluated for eligibility. Inclusion criteria were patients undergoing thyroid US for any thyroid indication, including athyreotic patients. Patients were excluded if they had suspected hyperparathyroidism, previous history of parathyroid disease, chronic kidney disease, post-thyroidectomy hypoparathyroidism, incidentally suspected parathyroid adenoma on US despite normal biochemical results, had undergone parathyroid surgery in the past year, or had histologically confirmed parathyroid tissue following thyroid surgery. Although routine calcium levels were not systematically measured on the day of the ultrasound for all patients, all participants were attending scheduled follow-up visits, allowing us to reliably exclude individuals with known or suspected parathyroid dysfunction based on existing clinical history and laboratory data.

### US examination and imaging analysis

US examination was performed by a physician with more than 20 years of experience in thyroid US and US-related procedures (PT). Bilateral thyroid lobes, isthmus, and various neck compartments were always examined in transverse and sagittal orientation. Patient were assessed as negative for normal PTG when they were not detected within 2–5 minutes of US exploration, depending on their initial indication. For ethical reasons, the images presumed to correspond to normal PTGs were not confirmed with cytological or histological samples.

### Defining a normal parathyroid gland

According to the literature and most of our experience, normal PTGs appear on US as small, oval or round structures, typically from 3 to 6 mm in major diameter. They exhibit slightly higher echogenicity compared to the adjacent thyroid tissue, with a typical homogeneous echotexture. They are closely adherent to the posterior aspect of the thyroid lobes, sometimes embedded within the thyroid parenchyma. Intrinsic vascularization is scant with a sort of hilum not always visualized. Parathyroid glands can be distinguished from lymph nodes by their more regular shape, higher echogenicity, and the absence of the central hyperechoic hilum which is characteristic of lymph nodes. Therefore, normal PTGs can be defined as round-to-oval shape, with homogeneous echostructure, mildly hyperechoic compared to normal thyroid parenchyma, and a potentially visible vascular hilum (5).

### Data collection

Regardless of their anatomical location, each identified normal PTG was recorded in a database, and its topography was evaluated to assess how the four typical PTGs could be mapped. To minimize biases, we chose to classify the glands based on their location rather than attempting to match them to one of the four specific PTGs, as it is not possible to determine this with certainty. PTG were searched around the thyroid gland, including the lower thyroid pole, upper thyroid pole, and the perithyroidal region (upper, middle, and lower thirds). Intra-thyroidal PTGs were excluded from the analysis to decrease bias. The dimensions of each recorded PTG, including anteroposterior (AP), length (L), and transverse (T) measurements, were recorded in the database, and the volume was calculated using the ellipsoid formula (AP × T × L × 0.52) and expressed in mm (3). The detection rate of normal PTGs was then calculated as the proportion of patients in whom at least one PTG was visualized relative to the total number of patients examined. In addition to imaging findings, patient general data (age, gender, and BMI) were collected. Clinical variables, including thyroid size and underlying thyroid disease, were also recorded. For statistical analysis, patients with euthyroid goiter or autonomous nodules were grouped into a single category, referred to as nodular disease. Nodular disease was defined as the presence of at least one nodule, while ultrasound features indicative of thyroiditis included hypoechoic areas with heterogeneous echotexture, echogenic septations, and lobulated margins. Thyroid enlargement was defined as a volume greater than 16 mL in males and 13 mL in females, based on the median thyroid volumes observed in our study population. These data were analyzed to explore potential correlations between the visibility of PTGs and patient characteristics.

### Statistical analysis

Descriptive statistics were calculated for continuous variables, such as age, BMI, and thyroid size, with results presented as median and interquartile range (IQR). Categorical variables, such as gender and the presence of thyroid pathologies, were summarized as frequencies and percentages. The relationship between the detection rate of PTGs and patient characteristics (e.g., BMI, thyroid pathology) was analyzed using Pearson’s correlation for continuous variables and chi-square tests for categorical variables. A p-value of less than 0.05 was considered statistically significant. All statistical analyses were conducted using Jamovi software version 2.3 (https://www.jamovi.org, Sydney, Australia).

## Results

### Characteristics of study population

**Figure 1:**
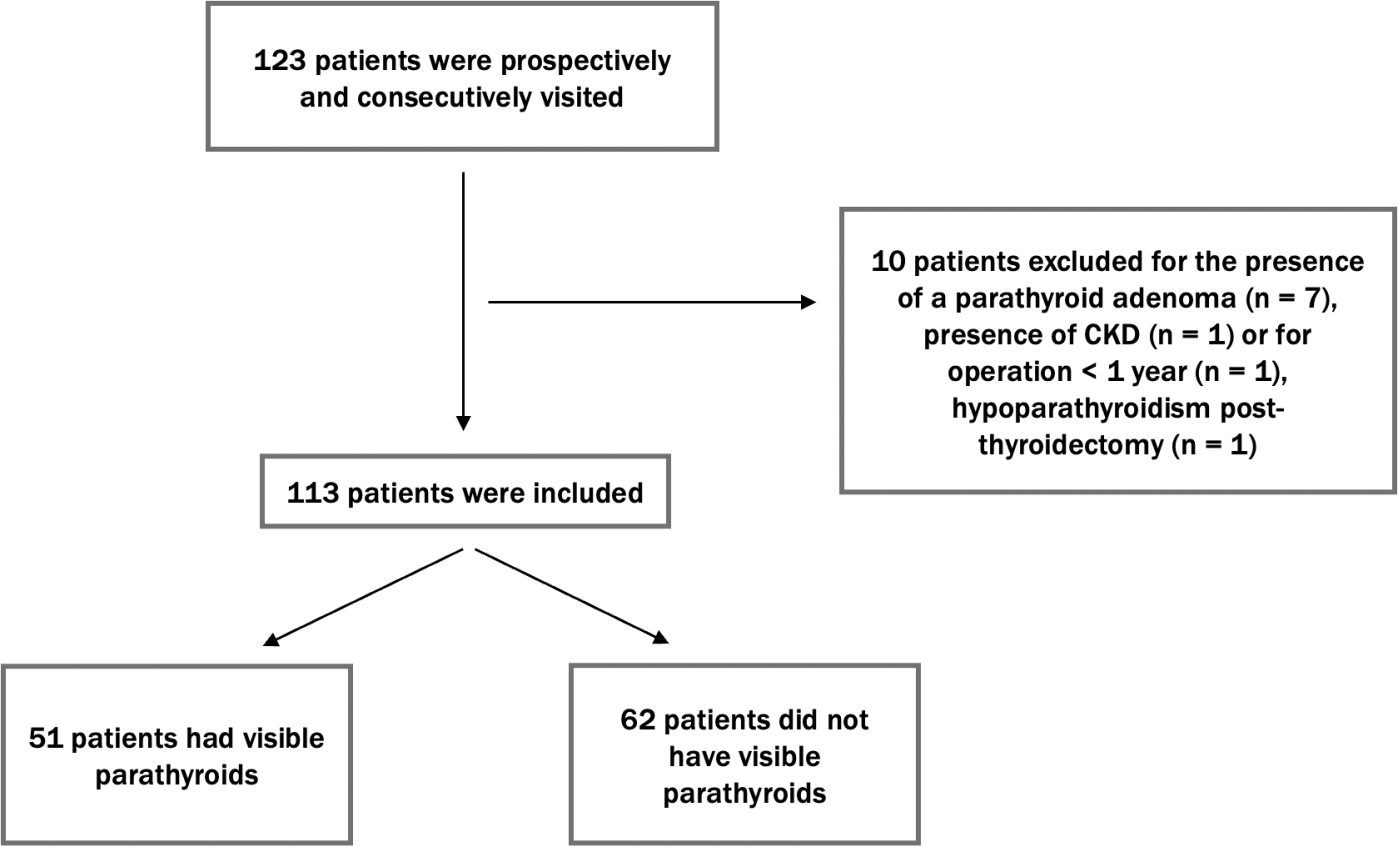
Flow diagram of patient enrollment. This diagram shows the selection process of 123 patients, with 10 excluded due to specific causes. The final 113 patients were examined for PTG visibility, with 51 showing visible PTGs and 62 having no visible PTGs.

According to the study design, 123 patients were initially considered, with 10 subsequently excluded for reasons detailed in **Figure 1**. Finally, 113 patients were enrolled in the study. Of these, 80 were female. The median age of the cohort was 55 (IQR: 46–64). The median body weight was 67 kg (IQR: 56-78), with a median BMI of 24.1 kg/m². Thyroid volume was shown to be correlated with BMI (p value <0.001). Based on their thyroid disorder, the patients were categorized into the following subgroups: 40 patients had thyroiditis, 52 had nodular disease, 1 had de Quervain’s thyroiditis, 10 had undergone total thyroidectomy, and 5 had undergone partial thyroidectomy. The median thyroid volume in patients who did not undergo total or partial thyroidectomy (n = 98) was 12 mL, while the volume of thyroid remnants in patients who had a hemithyroidectomy was 5 mL.

### Parathyroid detection

**Figure 2:**
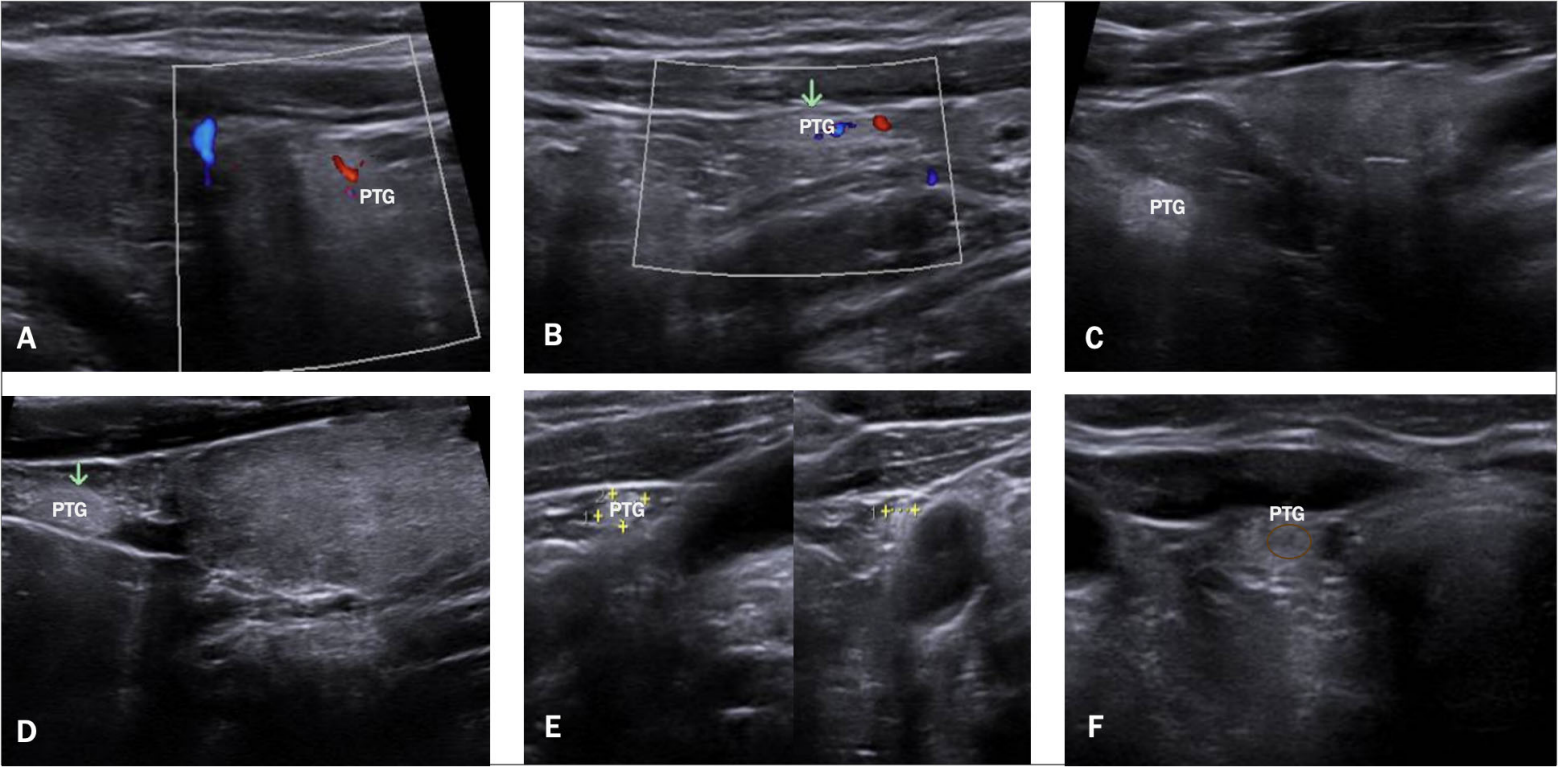
Ultrasound Images of PTGs. This figure displays a series of US images. **(A, B)** show a visible vascular hilum using color Doppler, **(C, D)** show normal PTGs with mild hyperechogenicity, **(E)** highlights the ectopic PTGs adjacent to the carotid artery, and **(F)** shows a normal PTGs with its visible hypoechoic hilum.

A total of 61 PTGs were identified in the study population, with 51 patients (45,1%) having at least one normal PTG detected. Specifically, 41 patients (80,3%) had only 1 PTG visualized, and 10 patients (19,7%) had 2 PTGs identified, while no patients had 3 or 4 PTGs. The median size of the PTGs was 2.8 mm (AP) x 3.3mm (T) x 4.8 mm (L); and the median volume was 23.7 mm (3). All PTGs were mildly hyperechoic compared to the normal thyroid gland and had a homogeneous structure. In most PTGs, the capsule was clearly visible while hilum was identified at Doppler in nearly all cases. **Figure 2** displays a series of PTG images collected during the study. The PTGs were detectable in both longitudinal and transverse views, although the initial and easier detection was typically in the longitudinal view.

### Topographic anatomy

**Figure 3:**
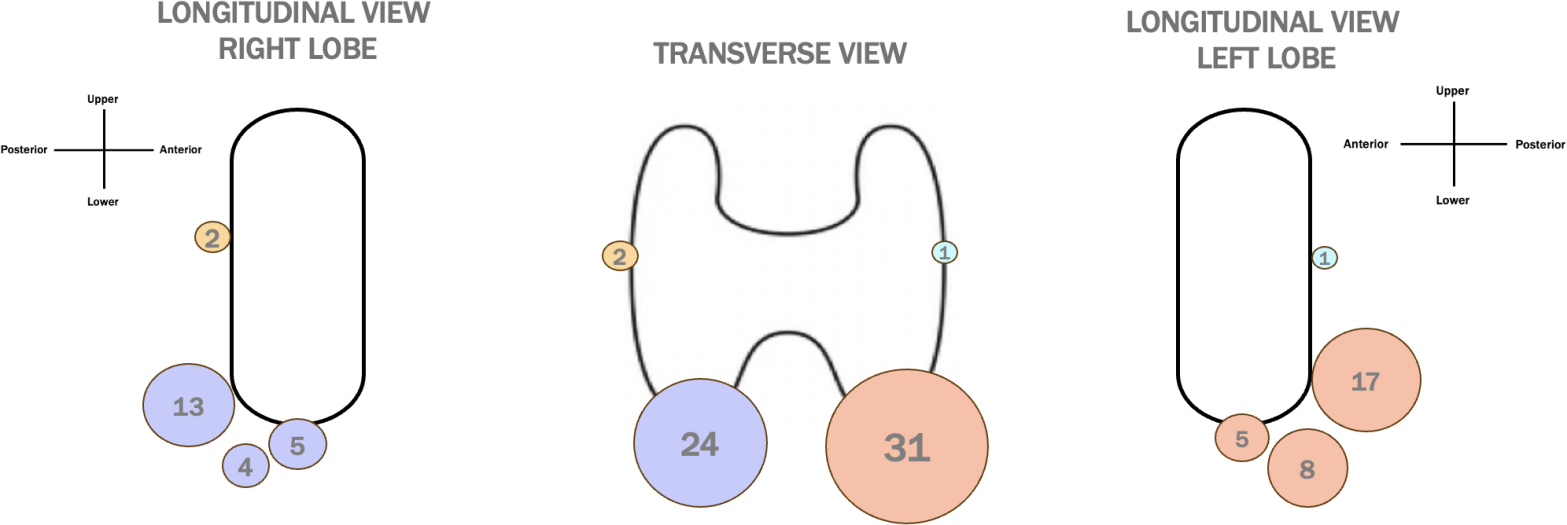
Distribution of PTG Locations in Patients with Thyroid. A transverse and longitudinal view of PTG locations in thyreotic patients, with dots of different dimensions representing the frequency of PTG detection. The colors correspond to specific anatomical locations: yellow for the right superior PTGs, purple for the right inferior PTGs, light blue for the left superior PTGs, and orange for the left inferior PTGs.

Regarding the location of PTGs in thyroid patients, 24 PTGs were identified as right and 31 as left. With respect to the right thyroid lobe, 2 PTGs were located in the posterior middle third peri-thyroid region, 13 in the posterior lower third perithyroidal region, and 9 directly beneath the lower lobe pole. Of the latter group, 5 PTGs were attached to the thyroid pole, while 4 were free from it. In the left lobe, 1 PTG was found in the posterior middle third, 17 in the posterior lower third, and 13 at the lower pole of the thyroid lobes; 5 PTGs were attached to the pole, while 8 were free from it. All the posterior PTGs were close to but not attached to the lobe pole. In athyreotic patients, 5 PTGs were identified in 10 patients. One ectopic PTG was found adjacent to the carotid artery. No intra-thyroid PTGs were considered. **Figure 3** illustrates the locations of PTGs in patients with thyroid tissue, both in frontal and longitudinal views.

### Analysis according to individual features

We further analyzed the data by dividing the patient population according to the thyroid disorders they presented with, and examining correlations with gender, median age, BMI, thyroid volume, and various parathyroid characteristics, such as the number of visualized PTGs and their mean volume. These correlations are detailed in **Table 1**. Next, we classified the population into two groups based on the visibility of PTGs: Group 1 (PTGs visible) and Group 2 (PTGs not visible). **Table 2** compares these groups in terms of thyroid volume and underlying thyroid pathology (thyroiditis, nodular disease, or athyreotic status). Our results indicate that the presence of thyroiditis or nodular disease does not significantly impact PTG visibility. On the contrary, thyroid volume and the inclusion of athyreotic patients were found to be statistically significant factors.

**Table 1:** Demographic and clinical characteristics by thyroid pathology.

Pathology	N. of patients	Gender F/M	Median Age (yrs)	Median BMI (kg/m2)	Median thyroid volume (ml)	N. Of patients with retrieved PTGs	Number of parathyroids visualized	Median parathyroid volume (ml)
Total study population	113	80/33	55	24,1	12	51	61	0.023
Thyroiditis	40	25/15	53	23	10	19	22	0.022
Nodular disease	52	41/11	56	24,6	13	26	30	0.024
Athyreotic	10	6/4	55,5	24,4	–	3	5	0.017
Partial thyroidectomy	5	4/1	60	26,2	5	3	4	0.041
SAT	1	0/1	46	24,7	12	0	0	–

This table summarizes patient demographics and thyroid volume, along with the number and volume of visualized PTGs across different thyroid conditions.

**Table 2:** Comparison of Patient Characteristics Based on PTG Visibility.

Parameter	Group 1	Group 2	p value
N. of patients	51	62	
Median thyroid volume (ml)	10	12	0,001
Patients with nodular disease	26	26	0,889
Patients with thyroiditis	19	21	0,069
Athyreotic patients	3	7	0,001

This table compares Group 1 (PTGs visible) and Group 2 (PTGs not visible) across several parameters, including median thyroid volume, presence of nodular disease, thyroiditis, and athyreotic patients. The corresponding p-values for each parameter are also shown.

## Discussion

Identifying normal PTGs is important in both clinical and surgical contexts, especially to prevent complications during thyroid surgeries and then preserve parathyroid function. The main aim of our study was to evaluate the detection rate of normal PTGs, assess their structural characteristics, map their most frequent locations, and investigate the factors influencing their visibility across various thyroid disorders.

In our study, PTGs were visible in 51 out of 113 patients, representing a detection rate of 45%. This is lower than detection rates reported in some other studies (2, 3, 6, 7), which may be partially explained by our decision to exclude intra-thyroid PTGs to minimize bias. This exclusion was necessary because differentiating a PTG from thyroid tissue in this region is nearly impossible and would require FNA for confirmation. **Table 3** provides a comparison of various studies in literature (2, 3, 6–9), highlighting differences in PTG detection rates, gland sizes, thyroid disorders of patients, and PTG locations.

**Table 3:** Comparative Ultrasound Characteristics of Normal Parathyroid Glands Across Studies.

Parameter	Marchand et al. (2024) (2)	Xia et al. (2019) (6)	Kim et al. (2024) (3)	Chen et al. (2023) (8)	Yoon et al. (9)	Wu et al. (2024) (7)	Present study
Study design	retrospective, consecutive	retrospective consecutive	retrospective consecutive	retrospective	retrospective, consecutive	retrospective	retrospective, consecutive
N. of patients studied	192	46	161	84	70	703	113
N. of PTG visible	197	106	294			1038	61
N. of patients with PG visible	144 (75%)	—	—	68 (81%)	67 (95,7%)	575 (81,79%)	51 (45%)
% only one PTG visible	98 (68%)	—	—	—	8 (11,4%)	235 (33,43%)	41 (80,3%)
% two PTG visible	40 (27,8%)	—	—	—	22 (31,4%)	242 (34,42%)	10 (19,7%)
% three PTG visible	5 (3,5%)	—	—	—	20 (28,6%)	73 (10,38%)	0
% four PTG visible	1 (0,7%)	—	—	—	16 (22,9%)	25 (3,56%)	0
Mean size							
Height	5,88mm	6,8mm	6mm	7,25mm	—	6,6mm	4,8mm
Width	4,05mm	4,6mm	3mm	4,65mm	—	4,8mm	3,3mm
Thickness	2,68mm	3,7mm	—	3,88mm	—	3,6mm	2,8mm
Mean volume	33,3mm3		—		—	62,1mm3	23,7 mm3
N. of patients with goiter with visible PG	38 (26,4%)	—	—	—	—	—	26 (23%)
N. of patients with thyroiditis with visible PG	31 (21,5%)	—	—	—	—	—	19 (16,8%)
% of population athyreotic with visible PG	—	—	—	—	—	—	3 (2,7%)
Location Lower pole	66 (33,5%)	9 (8,5%)	43 (17%)	—	—		21 (34,4%)
Right	28	—	—	—	—	—	9
Left	38	—	—	—	—	—	13
Upper pole	2 (1%)	—	8 (2,7%)		—		0
Posterior lower peri thyroid	27 (13,7%)	57 (53,7%)	234 (79,6%)	—	—	—	30 (49,1%)
Right	12	—	—	—	—	—	13
Left	15	—	—	—	—	—	17
Posterior middle peri thyroid	24 (12,2%)	—	—	—	—	—	3 (4,9%)
Right	14	—	—	—	—	—	2
Left	10	—	—	—	—	—	1
Infra-thyroid	64 (32,5%)	—	—	—	—	—	—
Other	14 (7,1%)	40 (37,8%)	8 (2,7%)	—	—	—	1 (1,6%)

This table outlines key findings on the visualization and features of normal PTGs as observed in several US studies. Data are categorized based on key findings, offering a comparative analysis of PTG imaging outcomes.

When analyzing factors that could influence PTG visibility, our data, as shown in **Table 2**, suggest that neither thyroiditis nor nodular disease significantly impacts PTG detection. This contrasts with the findings of Marchand et al. (2), who reported a significant association between these thyroid disorders and PTG visibility, noting that patients with goiter had less visible PTGs, while those with thyroiditis had more visible PTGs. Our results, however, highlight that thyroid volume is the most significant factor affecting PTG detection (p-value = 0.001). Thus, it appears that it is not the disease itself that affects PTG visibility, but rather thyroid volume, which may sometimes be linked to the thyroid disorder. Moreover, the thyroid gland is influenced by body weight; for example, obesity—particularly morbid obesity—has been associated with alterations in the thyroid ultrasound pattern (10). Additionally, thyroid volume positively correlates with BMI (11). This indicates that individuals with lower BMI often have smaller thyroids, which may facilitate PTG detection, while those with higher BMI tend to have larger thyroids, potentially impairing PTG visibility even without significant pathology. Currently, our study is one of the only to include athyreotic patients, as most research focuses on pre-operative cases (8, 9). Among the 10 athyreotic patients in our cohort, only 30% (n=3) had visible PTGs. This lower detection rate may be due to the fact that during thyroidectomy, PTGs may have been displaced, and the absence of the thyroid as a key anatomical reference point makes locating PTGs more challenging.

Although several studies describe normal PTGs, there is still a need for a clear definition of what constitutes a normal PTG on US. In our study, we based our assessment on both the characteristics of normal PTGs described in the literature—namely, mild hyperechogenicity and a homogeneous structure—and the typical features of parathyroid adenomas (5). We can confirm the presence of hyperechogenicity in normal PTGs, though not as pronounced as sometimes described. We suggest that this hyperechogenicity is often overstated, particularly in patients with conditions like thyroiditis, where the hypoechoic thyroid tissue makes PTGs appear relatively more hyperechoic. The reason why normal PTGs tend to appear hyperechoic, in contrast to parathyroid adenomas, is due to their higher fat content, which ranges from 10% to 50% (4). This variability in fat content among individuals may help explain why not all PTGs are consistently visible on US. Moreover, our study identified an important yet often understated feature: nearly all PTGs displayed a hypoechoic “vessel” that could also be visualized using Doppler (see **Figure 2**, image F), similar to what is observed in parathyroid adenomas (5). This vascular hilum was very important in our experience for distinguishing PTGs from surrounding tissues, despite not being frequently described in previous studies (2, 6).

Differentiating normal PTGs from other neck structures may be challenging. The differential diagnosis includes hyperechoic structures, due to fat content, such as lipomas, lipomatosis of lymph nodes, and epidermoid cysts (12, 13). However, these entities represent relatively uncommon findings in the neck as compared to the likelihood of finding normal PTGs. Both metastatic and benign lymph nodes should also be considered. Metastatic lymph nodes, particularly those from papillary thyroid carcinoma, may exhibit focal or diffuse hyperechogenicity, but they are typically larger, irregular in shape, and have a heterogeneous echotexture (14). In contrast, benign lymph nodes usually display a central hyperechoic hilum — a key feature that distinguishes them from normal PTGs, which have a vascular hilum lacking hyperechoic characteristics.

Our study also investigated the anatomical distribution of PTGs. Typically, there are four PTGs, each with distinct embryological origins. The superior PTGs undergo a shorter embryological migration, and as a result, their final position is generally stable, usually located on the posterolateral surface of the middle to superior thyroid lobe. In contrast, the inferior PTGs originate from the third branchial pouch, and their longer migration path increases the likelihood of ectopic localization. Inferior PTGs are often situated on the posterolateral surface of the lower part of the thyroid lobe (15). In our study, the majority of PTGs were located at the lower thyroid pole, with a significant number found in the posterior perithyroidal region, most likely corresponding to the inferior PTGs (orange and blue dots in **Figure 3**). Only a small number of PTGs were detected in the posterior middle third, likely representing the superior PTGs (blue and yellow dots in **Figure 3**). This finding aligns with previous studies, which also reported that inferior PTGs are more commonly identified than superior ones (2, 3). Interestingly, this observation contrasts with intraoperative findings, where superior PTGs are more readily identified by surgeons (16). This discrepancy may be related to the more stable and consistent anatomical position of superior PTGs during surgery. In preoperative US, however, superior PTGs may be more susceptible to distortion and compression by surrounding dense tissues, such as fat and muscles, making them more difficult to detect with ultrasonography (16).

The present data can be discussed from the surgeons’ point of view. The upper parathyroids have a “classic” position in 80% of cases while the inferior ones in only 50%. In addition, surgeons must take under consideration the possibility of ectopic, supra- or sub-numerary PTGs (16–18). Having the possibility to know where to find normal PTGs before surgery can certainly help the surgeon in their identification and thus reduce their accidental lesion or removal, especially in patients undergoing thyroidectomy plus central cervical dissection (19). The low detection rate of PTGs in the cases previously thyroidectomized can be explained by the fact that once the thyroid is removed the PTGs often retract deeper into the tissue. In any case, a possible atypical position of PTGs should also be considered.

From the embryological standpoint the easier US detection of inferior PTGs can be due to the presence of thyreo-thymic ligament, above which there is the thyreo-thymic extension (i.e., a variable projection of thyroid tissue coming from the lower pole) (20). As it is known how the surgical dissection of the thyreo-thymic ligament helps in maintaining the inferior PTGs integrity avoiding iatrogenic damage during thyroidectomy (21), similarly it may be hypothesized that the low echogenicity of the connective tissue of thyreo-thymic ligament delimits an “echographic lodge” which hosts the inferior PTGs allowing their detection. On the contrary, the easier detection of superior PTGs during surgery is justified by the shorter embryological migration to come from the dorsal wing of the IV pharyngeal pouch together with the ultimo-branchial body (which will give rise to the thyroid parafollicular cells). Instead, their difficult US detection can be due to their topographic anatomy; their location at the laryngeal crycho-thyroid articulation, 1 cm above the intersection between the inferior laryngeal nerve and the inferior thyroid artery (22), may be affected by “acoustic interferences” (due to the described structures) disturbing the US signal. Additionally, while positional anomalies are less common in superior PTGs compared to inferior ones, they may occasionally result in retro-laryngeal or retro-esophageal locations, further complicating their detection.

Overall, this study gains importance because preoperative planning is being increasingly discussed. Since US is the most accurate method for evaluating the neck region, identifying PTGs can help reduce the already rare cases of postoperative hypoparathyroidism. US is particularly useful because, unlike scintigraphy, which only identifies hyperfunctioning tissue (23), it can also detect normal PTGs that would otherwise be missed.

A limitation of our study is that all PTGs were evaluated by a single operator, which may introduce some degree of operator-dependent bias. Also, it is important to consider the possibility of ectopic PTGs (24), which suggests that our detection rate may be underestimated, as even eutopic PTGs can occasionally be challenging to visualize. Furthermore, even if we systematically attempted to identify PTGs, their detection is also influenced by the individual neck anatomy. On the other hand, the strengths of our study include demonstrating that PTGs can be identified when systematically searched for in a consecutive cohort. We also minimized potential bias by excluding intrathyroidal PTGs from the analysis and included a population with highly heterogeneous thyroid disorders.

In conclusion, normal PTGs can be visualized using US. In our study, 51 out of 113 patients had at least one normal PTG visible. The easiest locations to detect PTGs with US are the regions near the lower pole, either posteriorly or just below, in line with the pole. The presence of thyroid disease has minimal impact on US sensitivity. In patients with large thyroids or in those who are athyreotic, PTGs are rarely found. Clinical endocrinologists, radiologists, and surgeons should be aware of these findings.

## Data Availability

The raw data supporting the conclusions of this article will be made available by the authors, without undue reservation.
