# Correction: Ultrasound detection of normal parathyroid glands: detection rate, topographic anatomy and the role of underlying thyroid disease

**DOI:** 10.3389/fendo.2025.1664368

**Published:** 2025-08-12

**Authors:** Isabella Chiardi, Petra Makovac, Andrea Leoncini, Flavio Forte, Mario Rotondi, Pierpaolo Trimboli

**Affiliations:** ^1^ Department of Internal Medicine and Therapeutics, University of Pavia, Pavia, Italy; ^2^ Thyroid Unit, Clinic for Endocrinology and Diabetology, Ente Ospedaliero Cantonale (EOC), Bellinzona, Switzerland; ^3^ Service of Surgery, Ospedale Regionale di Mendrisio, Ente Ospedaliero Cantonale (EOC), Mendrisio, Switzerland; ^4^ Clinic for Radiology, Imaging Institute of Southern Switzerland, Ente Ospedaliero Cantonale (EOC), Bellinzona, Switzerland; ^5^ Urologic Division, Vannini Hospital, Rome, Italy; ^6^ Istituti Clinici Scientifici Maugeri IRCCS, Unit of Endocrinology and Metabolism, Laboratory for Endocrine Disruptors, Pavia, Italy; ^7^ Faculty of Biomedical Sciences, Università Della Svizzera Italiana (USI), Lugano, Switzerland

**Keywords:** US, normal parathyroids, parathyroid detection, parathyroid imaging, neck ultrasound

In the published article, there was an error in the **Discussion** section regarding the citation of the article by Marchand et al. Our article incorrectly referred to “64 intra-thyroid parathyroids” instead of “infra-thyroid.” Accordingly, the authors have removed this citation from the relevant passage (while keeping it elsewhere as it is cited multiple times), since the citation was no longer appropriate.

A correction has been made to the section **Discussion**, Paragraph 2:

“In our study, PTGs were visible in 51 out of 113 patients, representing a detection rate of 45%. This is lower than detection rates reported in some other studies (2, 3, 6, 7), which may be partially explained by our decision to exclude intra-thyroid PTGs to minimize bias. This exclusion was necessary because differentiating a PTG from thyroid tissue in this region is nearly impossible and would require FNA for confirmation. Table 3 provides a comparison of various studies in literature (2, 3, 6–9), highlighting differences in PTG detection rates, gland sizes, thyroid disorders of patients, and PTG locations.”

The original version of this article has been updated.

